# Our Second Hexade

**Published:** 1833

**Authors:** 


### III. ORIGINAL.

### The Western Journal.

E SYLVIS, AEQUE ATQUE AD SYLVAS, NUNCIUS.

*Cincinnati, October* 1, 1833.

### I. OUR SECOND HEXADE.

We may perhaps be pardoned for saying, that the plan of commencing a second hexade, meets the approbation of the profession in the West, as has been evinced by a very considerable number of subscribers since our last number was issued. We shall of course find in this encouragement, a new stimulus to exertion; but as it is impossible for a humble individual, immersed in the cares of professional business, to give variety and richness to a medical journal of 600 pages *per annum*, we again beg leave, respectfully to solicit our western brethren, to send us reports of their experience, that they may be made mutually to instruct each other.

